# Bioinspired Design of Ergonomic Tool Handles Using 3D-Printed Cellular Metamaterials

**DOI:** 10.3390/biomimetics10080519

**Published:** 2025-08-08

**Authors:** Gregor Harih, Vasja Plesec

**Affiliations:** Laboratory for Integrated Product Development and CAD, Faculty of Mechanical Engineering, University of Maribor, Slovenia Smetanova ulica 17, 2000 Maribor, Slovenia; vasja.plesec@um.si

**Keywords:** bioinspired design, product ergonomics, 3D printing, tool handle, finite element method, user comfort, cellular metamaterials

## Abstract

The design of ergonomic tool handles is crucial for user comfort and performance, yet conventional stiff materials often lead to uneven pressure distribution and discomfort. This study investigates the application of 3D-printed cellular metamaterials with tunable stiffness, specifically gyroid structures, to enhance the ergonomic and haptic properties of tool handles. We employed finite element analysis to simulate finger–handle interactions and conducted subjective comfort evaluations with participants using a foxtail saw with handles of varying gyroid infill densities and a rigid PLA handle. Numerical results demonstrated that handles with medium stiffness significantly reduced peak contact pressures and promoted a more uniform pressure distribution compared to the stiff PLA handle. The softest gyroid handle, while compliant, exhibited excessive deformation, potentially compromising stability. Subjective comfort ratings corroborated these findings, with medium-stiffness handles receiving the highest scores for overall comfort, fit, and force transmission. These results highlight that a plateau-like mechanical response of the 3D-printed cellular metamaterial handle, inversely bioinspired by human soft tissue, effectively balances pressure redistribution and grip stability. This bioinspired design approach offers a promising direction for developing user-centered products that mitigate fatigue and discomfort in force-intensive tasks.

## 1. Introduction

The rapid evolution of handheld products and tools driven by technological advancements has not been matched by equivalent progress in their physical interaction with users. Consequently, research efforts have been directed toward augmenting human tactile perception through the application of various ergonomic and biomechanical principles. Much of this research has concentrated on enhancing grip strength, minimizing muscle exertion, and improving safety and comfort by optimizing the diameter of cylindrical handles [1,2,3,4,5,6]. Gripping a cylindrical handle typically results in uneven contact pressure, which can lead to discomfort, pain, and the development of musculoskeletal disorders [7].

Research indicates that grip strength is influenced by anthropometric factors, with larger hand dimensions correlating with greater strength, while individual finger force also increases with anthropometric growth [8]. Additionally, the handle shape affects grip force, with elliptic handles reducing force by 7% compared to circular ones [9]. Muscle coordination varies with handle shape, which may affect ergonomics and injury risks, such as tendonitis and lateral epicondylalgia. Researchers suggested that product handles should conform to the hand’s natural grasp posture to improve comfort, safety, and performance [10]. Non-cylindrical handles, like ellipsoidal and triangular shapes, reduced grip variability, providing a more stable and comfortable grip [11].

Advancements in digital human hand modeling have facilitated the identification of handle shapes with enhanced ergonomics [12,13]. However, achieving size and shape optimization for a broad target population is hindered by the inherent variability in hand anthropometric dimensions among users, including hand/finger length, hand width, and finger circumference [8]. Consequently, accommodating all users with a single-sized handle is impractical, leading to suboptimal gripping characterized by high contact pressure on soft tissues, increased muscle activity, diminished stability, performance, and safety for many individuals [5]. Moreover, companies typically avoid offering multiple handle sizes to mitigate hand size variation [14].

To improve the ergonomics of product handles, researchers have studied the effects of contact pressure during gripping and established threshold values for pressure discomfort (PDT) and pressure pain (PPT) through both objective measurements and subjective assessments [15,16,17]. Findings indicated significant individual variability in these thresholds. Specifically, an average PDT of 188 kPa was determined for the finger area, 200 kPa for the palm, and 100 kPa for the thenar region. The corresponding average PPT values were 496 kPa for the fingers, 494 kPa for the palm, and 447 kPa for the thenar area [18]. It has also been shown that contact pressure on the hand is a better predictor of gripping comfort than finger joint angles [19]. For cylindrical handles, measuring contact pressures in key areas of the hand, such as the fingertips, distal palm, and proximal palm is sufficient to evaluate overall grip comfort.

The soft tissue of the human hand, which includes skin, subcutaneous tissue, muscle, fascia, and others, is highly deformable and exhibits nonlinear mechanical behavior under stress. It shows low stiffness at smaller strains and a rapid increase in stiffness at higher strains [20]. Consequently, as the grip forces increase, contact pressures rise sharply due to tissue deformation. This relationship underscores the importance of optimizing handle design to minimize discomfort and potential injury.

Research has shown that deformable handles can enhance comfort when using handheld products. Understanding the biomechanical interaction between the hand and the handle, particularly in terms of the handle material’s mechanical response, is crucial for optimizing handle design beyond size and shape. However, much of the research to date has focused on stiff handle materials such as steel, plastic, and wood, overlooking the potential benefits of more deformable materials. Most modern handheld product handles are made from plastics with rubber coatings, which increase friction but remain significantly stiffer than the soft tissues of the hand [21]. As a result, most of the deformation occurs in the hand, leading to high contact pressure values.

In response, researchers have investigated elastomeric foam materials, which reduce the overall contact pressure and improve the pressure distribution, thus enhancing comfort [22]. However, materials that are too soft or too thick can compromise handle stability. The optimal solution appears to involve materials that remain stable under lower gripping forces and only deform when critical pressure levels are reached [23]. This highlights the importance of careful material selection in ergonomic design, a factor that is often overlooked in the design and analysis of product handles.

Furthermore, measuring peak contact pressures and pressure distribution at the hand–handle interface is extremely challenging due to the complex shape of the hand and the small contact areas at the fingers, which cannot be accurately captured with most pressure-sensitive films or pressure mapping systems [24,25,26,27]. To address this limitation, the finite element method (FEM) has been used in biomechanics and ergonomics to identify and optimize parameters influencing handle design [28,29,30,31,32,33,34]. In previous studies, we employed the FEM to model and simulate hand–handle interactions using different materials, enabling us to identify those that improve comfort [35]. Three-dimensional printing technology has been used to fabricate deformable materials for testing subjective responses [36]. Nevertheless, optimizing material properties to reduce peak and overall contact pressures while maintaining handle stability remains a complex task due to the intricate nature of the hand–handle biomechanical system.

Previous research suggested that a combination of objective measurements and subjective assessments is crucial for comprehensive tool evaluation and design optimization [37]. The study also confirmed that the handle shape significantly influenced hand grip effort, usability, and discomfort levels, consistent with prior research.

To address the complexities and interdependencies of material properties, contact mechanics, and human perception, the objective of this study is to investigate the application of 3D-printed cellular metamaterials with tunable stiffness in ergonomic tool handles. This is achieved by developing a finite element model to predict finger–handle contact mechanics and correlating these numerical findings with subjective comfort evaluations, thereby establishing a comprehensive understanding of how material properties influence haptic performance and user comfort.

## 2. Materials and Methods

### 2.1. Handle Material Properties

**Figure 1:**
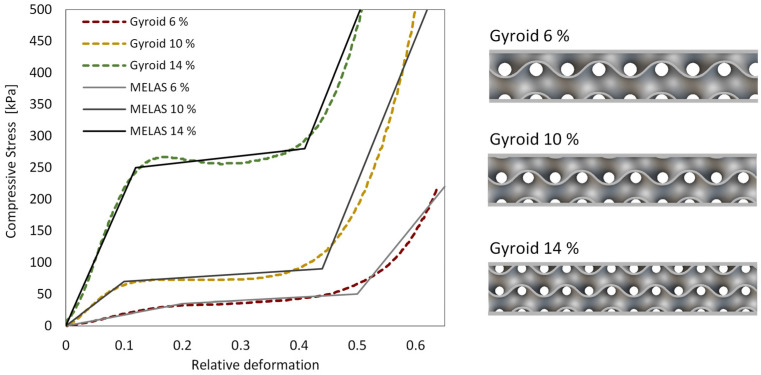
Compressive stress [kPa]–relative deformation [-] graph derived from experimental data for gyroid structures with densities of 6%, 10%, and 14%, along with their respective approximated multilinear elastic material models (**left**). Additionally, a graphical representation of the gyroid structures ranging from 6% to 14% density, depicted from top to bottom (**right**).

**Material model of cellular structures:** The deformable samples were fabricated using theFused Filament Fabrication (FFF) 3D printer Creality CR-10 4S. Thermoplastic Polyurethane (TPU) with a Shore hardness of 95A was selected (Azurefilm 95A, Azurefilm, Slovenia) as the material for the handle, featuring a gyroid metamaterial cellular structure to achieve the desired deformability of the interface. A notable advantage of the gyroid metamaterial structure is its near-isotropic mechanical response, which is essential in tasks where forces are applied in multiple directions [38]. The infill density, which denotes the proportion of internal volume occupied by solid material compared to the total volume of the structure, varied. Specifically, 6%, 10%, and 14% densities were chosen based on prior examination and analysis, providing distinct responses corresponding to soft, medium, and hard structural characteristics. The TPU handle was 3D-printed using two perimeters in the outer and inner wall, while the gyroid structure was printed using one perimeter. To characterize the stress–strain response of the material, experimental procedures followed the ISO 3386-2:1997 standard, with the specimen height being adjusted to match the final deformable layer thickness of the handle (Figure 1).

To ensure a stable numerical representation of the mechanical behavior of the 3D-printed gyroid structures, a multilinear elastic material model (MELAS) was used. This approach approximates the mechanical response of the cellular structure without directly incorporating its intrinsic geometry, thereby ensuring numerical stability during finite element analysis (FEA). Manual fitting of stress–strain curves was conducted using three distinct slopes per material: the initial slope corresponding to the structure’s stiffness, followed by the plateau region, and finally, the steep slope indicating the densification phase of deformation. Significant differences in stiffness between the 6%, 10%, and 14% density structures were observed, highlighting the potential to design cellular structures for desired mechanical properties selectively.

**The 3D-printed plastic material model:** A 3D-printed poly (lactic acid) (PLA) material model, representing commodity plastic, was investigated along with the flexible TPU gyroid structure, contrasting the rigidity of PLA with the compliant nature of soft tissue. The PLA handle was printed with 100% infill to ensure maximum stiffness and structural safety. Its inclusion allowed for identical geometries to be maintained across all handle types, thereby eliminating shape-related variability and ensuring that any differences in user response could be attributed solely to material properties.

**Figure 2:**
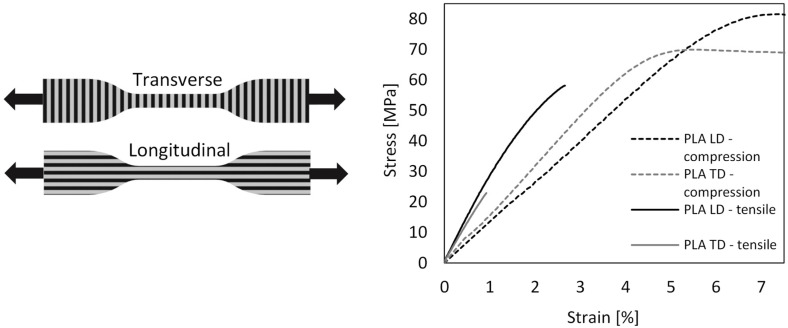
Schematic illustration of dumbbell specimens used in the uniaxial tensile test (**left**), alongside a stress–strain graph displaying the average outcomes from five samples per test (**right**).

A comparison between the flexible TPU and PLA handles assessed the impact of compliant material on reducing contact pressure and maintaining stability during grasping. To characterize the 3D-printed PLA, uniaxial tensile (ASTM D638-15) and compression (ASTM D695-15) tests were performed on transversely and longitudinally printed samples. Figure 2 shows the average stress–strain results and sample layer orientation. Experimental data gave a Young’s modulus of 2952.8 and Poisson’s ratio of 0.33, which were used in the linear–elastic material model for simulation.

### 2.2. Biomechanical Finger–Handle Numerical Model

**Figure 3:**
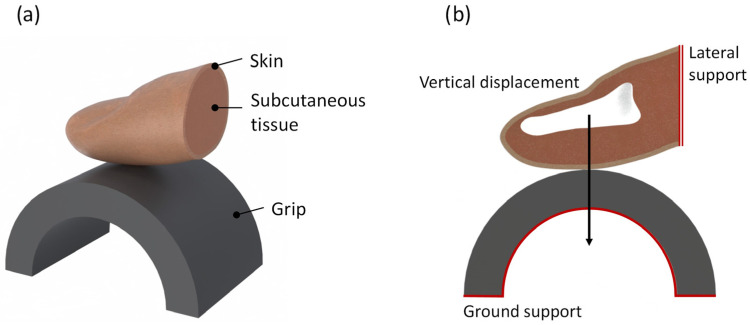
(**a**) Geometrical and (**b**) boundary conditions of the FE digital human finger model.

**Geometry and boundary conditions:** The geometric structure of the numerical finger model was derived from a previous study utilizing CT scans [39]. This anatomically accurate model encompasses the index finger’s tip, including skin, subcutaneous tissue, and bone layer. As reported in the literature, fingertip skin’s thickness typically ranges from 0.1 to 0.7 mm [40]. Therefore, a thickness of 0.7 mm was selected to ensure numerical stability and accommodate larger finite elements effectively. The distal phalanx was represented as a rigid cavity, as depicted in Figure 3b in the sectional view (white color).

The handle was modeled as a semi-cylinder with dimensions of 5.5 mm thickness and 36 mm diameter, aligning with ergonomic recommendations for handle diameter to facilitate optimal handle grip, maximizing finger force and subjective comfort [41]. The finger was positioned above the handle, oriented in a manner resembling the initial stance for a grasping task. During simulation, the inner circle and lower portion of the cylinder were fixed directionally and rotationally. Vertical displacement was constrained to the rigid cavity of the distal phalanx in 1 mm increments. Additionally, lateral support was provided to the proximal part of the modeled finger, allowing only for vertical displacement. Figure 3a displays the geometry of the numerical model with annotated components, while the loading conditions are illustrated in Figure 3b.

**Material properties and numerical mesh of the biomechanical model:** To account for the nonlinear behavior inherent in biological tissue, a hyperelastic material model is necessary. Therefore, the Ogden 3rd-order hyperelastic material model, as established in our prior research, was selected for defining the mechanical behavior of the subcutaneous tissue and skin [23]. Recognizing the significantly higher stiffness of the distal phalange bone, the bone was simplified and modeled as a rigid cavity to streamline the finite element count and reduce numerical complexity. Material properties of the PLA handle were defined using a linear–elastic model, while the MELAS material model was employed to define the properties of gyroid structures, as detailed in the preceding subsection. A comprehensive overview of the material models and their respective parameters for all components is provided in Table 1.

**Figure 4:**
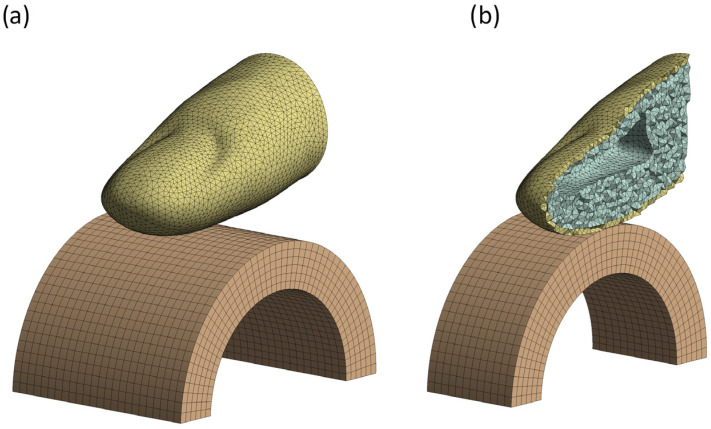
Meshed numerical model of finger and handle: (**a**) whole model; (**b**) section view.

The finger geometry was meshed using quadratic 10-node tetrahedral elements (Tet10), while the handle used quadratic 20-node hexahedral elements (Hex20), resulting in 86,010 elements with 154,573 nodes. Element size was optimized for accuracy and computational efficiency through mesh convergence analysis. Since further reduction showed no significant changes, an element size of 0.7 mm was used for the subcutaneous tissue and skin, while 1 mm was applied to the handle for numerical stability (Figure 4). A bonded contact was specified between the skin and subcutaneous tissue, while a frictional contact with a coefficient of 0.5 was defined between the skin and handle [32].

**Finite element analysis setup and simulation procedure:** A static implicit finite element analysis was performed using ANSYS Workbench (ANSYS, Inc., Canonsburg, PA 15317, USA) on a high-performance computer system featuring a Xeon CPU E5-2670 (2.60 GHz) processor with 16 cores, situated at the Faculty of Mechanical Engineering, University of Maribor. The simulation process was carried out in multiple steps, each subdivided into a minimum of 20 sub-steps. The simulation was conducted in 1 mm increments of vertical displacement until the reaction force at the stationary segment of the handle reached 35 N, which corresponds to the maximum force exerted by the index finger’s distal phalanx during a gripping task [2]. Analysis was performed at this point. The numerical results for finger–grip contact pressure, reaction force, and vertical displacement are presented in the Results Section.

### 2.3. Task Definition and Subjective Comfort Questionnaire

**Figure 5:**
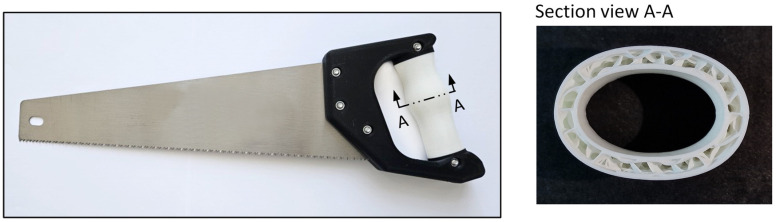
Foxtail saw used in the experiment with a deformable handle (**left**) and a sectional view of the gyroid metamaterial deformable handle during the printing process (**right**).

In this experiment, the chosen activity involved cutting wood with a foxtail saw, which demands a secure power grip and encompasses various hand-loading scenarios, including pushing, pulling, and twisting the tool using the tool handle. These loading patterns mirror those encountered in numerous everyday manual tasks utilizing handheld tools. Deformable handles integrating gyroid metamaterial structures, as detailed in the previous subsection, were fabricated using the FFF 3D printing technique with flexible TPU filament. The thickness of the cellular metamaterial structure remained uniform along the entire length of the handle. The foxtail saw used in the experiment, along with a sectional view showing the gyroid structure during the printing process, is presented in Figure 5.

Ten participants, spanning both genders, with an average age of 24, and meeting our health criteria, were selected. The mean hand size of subjects was 188 mm (SD 10 mm), aligning with the 50th percentile from the NHANES database. The experimental design for this study involved low-risk activities associated with normal tool usage. The study was conducted in accordance with institutional ethical standards and the principles outlined in the Declaration of Helsinki, ensuring respect for participants’ rights and safety. Additionally, the experimental procedure and protocol were approved by the ethics committee of the Faculty of Mechanical Engineering, University of Maribor. All participants were fully informed about the study’s objectives and procedures, and written consent was obtained prior to participation. Subjects used saws with different handles in a randomized order to make five cuts each, sawing 40 mm deep into 50 × 80 mm spruce wood at a comfortable, self-selected pace. A one-minute rest period was provided after each cut to minimize fatigue. All subjects were able to complete the task successfully, with no damage sustained to the handles or saws and no injuries reported. Afterwards they completed a subjective comfort rating questionnaire for each handle (1 = uncomfortable; 7 = comfortable), adapted from Kuijt-Evers, Vink [3], where they evaluated specific comfort descriptors: Fits good in the hand, Provides good force and torque transmission, Allows high task performance, Low grip force is needed for stable grip, Results in high peak contact pressures. Additionally, subjects needed to respond in terms of the overall comfort for each handle.

## 3. Results

### 3.1. Biomechanical Finger–Handle Numerical Model

**Figure 6:**
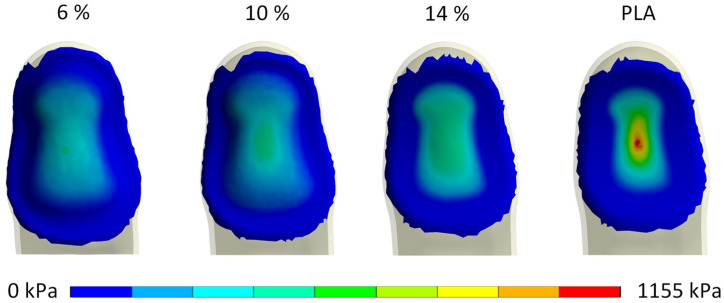
Contact pressure at handle–finger interface for all materials, normalized to the maximal contact pressure of the PLA structure.

**Contact pressure:** Apart from assessing the maximum contact pressure, the distribution of contact pressure plays a crucial role in the biomechanical and ergonomic evaluation of handle materials. Figure 6 illustrates the contact pressure between the finger and the handle for all considered handle materials. The pressure values were normalized based on the maximum contact pressure of the PLA handle, which was recorded at a reaction force of 35 N, measuring 1155 kPa.

**Figure 7:**
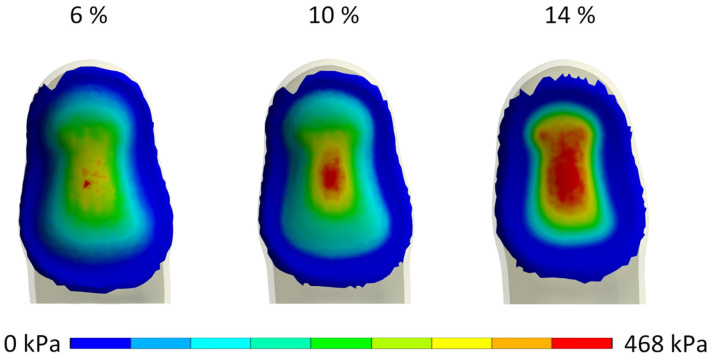
Contact pressure at handle–finger interface for cellular materials, normalized to the maximal contact pressure of a 14% dense gyroid structure.

Given the significant difference in contact pressure between the PLA handle and the gyroid structure handle, the pressure distribution appears notably similar for the gyroid handles. Consequently, Figure 7 focuses solely on the evaluation of gyroid structures, with pressure normalization based on the maximum pressure recorded with the 14% dense gyroid structure at the reaction force of 35 N, totaling 468 kPa.

**Figure 8:**
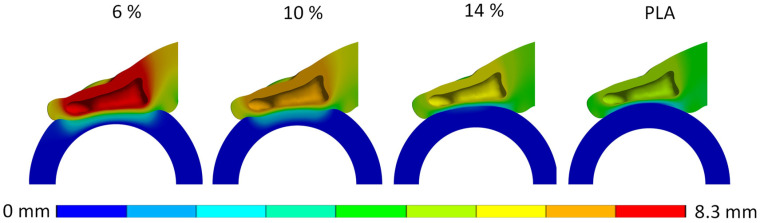
Lateral section view of the vertical displacement of the finger and the handle for all investigated structures, normalized to the maximal displacement of the 6% gyroid structure.

**Figure 9:**
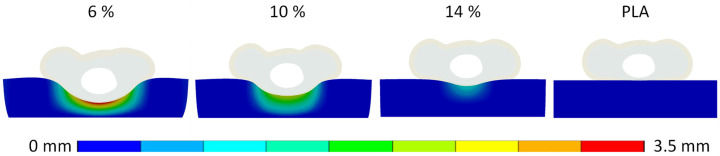
Anterior section view of the vertical displacement of the handle for all investigated structures, normalized to the maximal displacement of the 6% gyroid structure.

**Displacement:** The second parameter considered in the numerical evaluation of the handle structures was vertical displacement of the finger during grasping simulation. This evaluation included analyzing the vertical displacement for both the distal phalanx (Figure 8) and the handle structure (Figure 9). In both cases, the graphical representations were obtained at a reaction force of 35 N and normalized against the maximum displacement. Notably, the 6% dense gyroid structure exhibited the maximum displacement in both scenarios.

**Figure 10:**
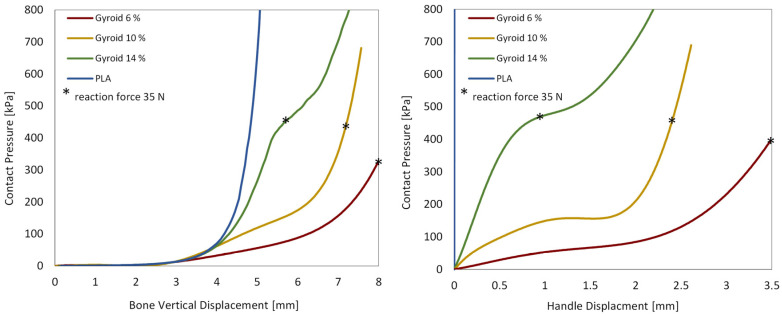
Contact pressure vs. bone vertical displacement graph (**left**) and contact pressure vs. handle displacement graph (**right**). Asterisk symbols indicate point where 35 N reaction force is reached.

**Figure 11:**
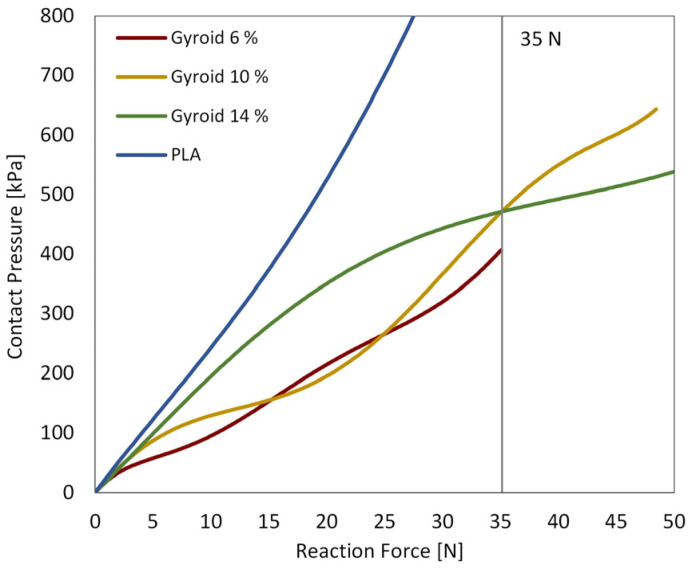
Contact pressure vs. reaction force. The vertical line represents a reaction force of 35 N, approximately the maximal force produced during a grasping task on the distal phalanx of the index finger.

**Contact pressure vs. displacement and reaction force:** The vertical displacement of both the phalanx and the handle was compared to the contact pressure, as shown in the following diagrams (Figure 10). In addition, the reaction force exerted on the stationary part of the handle was compared with the contact pressure between the fingertip and the handle (Figure 11).

### 3.2. Subjective Comfort Rating

**Figure 12:**
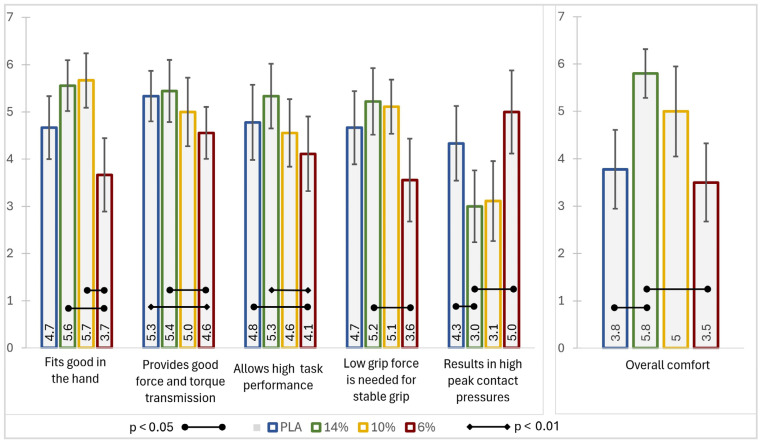
Findings indicating variations in comfort levels for both specific comfort descriptors and overall comfort, highlighting statistically significant differences among the handles.

Once the data from the subjective comfort questionnaire had been reported by the subjects, it was gathered and underwent processing, facilitating the calculation of mean values and corresponding standard deviations. To ascertain whether statistically significant differences existed among the comfort descriptors and overall rating across saws with different handles, a *t*-test for dependent samples was executed. We used a significance threshold of *p* < 0.05, commonly accepted as the standard for statistical significance, and reported results with *p* < 0.001 to indicate a stronger level of significance. Results from the subjective comfort rating questionnaire are presented in Figure 12, which allowed for a comprehensive assessment and discussion of the findings of subjective comfort ratings, along with a comparison to the numerical results.

## 4. Discussion

Balancing comfort and stability in handheld products is critical. Previous research has shown that soft handles can reduce peak contact pressure through deformation, thus improving comfort, but this may sacrifice stability if overly compliant. Conversely, stiff handles maintain stability but can cause discomfort due to higher localized pressures. Our approach leverages deformable gyroid structures exhibiting an inverse bioinspired mechanical behavior, where they remain stiff under low loads and deform only beyond a pressure threshold, thus combining the benefits of pressure redistribution without compromising stability. This novel design concept offers new insights for ergonomic handle development and haptic response.

**Influence of handle stiffness on contact pressure, deformation and subjective comfort:** Our results showed that the contact pressure between the finger and the handle varied significantly, with the highest pressure obtained with stiff plastic handles and the lowest with soft deformable handles, which was expected. When observing the contact pressure distribution, it is evident that stiff handle materials, such as PLA plastic, result in uneven, concentrated peak contact pressure. The peak contact pressure value was obtained at the location where the finger’s skin and subcutaneous tissue deform the most due to the contact with the handle and applied finger force. The plateau-like mechanical behavior of the cellular deformable handles resulted in considerably lower peak contact pressure values, as well as more uniform contact pressure distributions, due to the deformation of the handle material during the simulated gripping task.

Specifically, the contact pressure with the softest deformable handle material was 379 kPa at a reaction force of 35 N, indicating its ability to distribute contact pressure more uniformly. However, the soft deformable material also exhibited the highest deformation, reaching 3.47 mm, which compromised the stability of the handle in hands. Conversely, the medium and hard deformable handles demonstrated significantly lower contact pressures compared to traditional plastic handles (1155 kPa), with measured values of 454 kPa and 460 kPa, respectively, at a reaction force of 35 N. Notably, the hard deformable handle material exhibited low and controlled deformation, resulting in improved stability while maintaining relatively low contact pressures.

This has also been confirmed when observing the results from the subjective comfort rating descriptor “Low grip force is needed for stable grip.” The 14% dense gyroid structure handle was rated the highest and was statistically significant different from the 6% dense handle. Results from other handles were not statistically different, indicating no difference between them regarding this subjective comfort descriptor.

When evaluating the results from the subjective comfort rating, either the 14% or the 10% dense handle received the highest ratings for comfort predictors describing fit of the handle in the hand, simplicity of use, and providing good force and torque transmission, indicating that users found these handles to be the most comfortable in these aspects. The 6% dense handle consistently received lower ratings, especially with the comfort descriptor “Fits well in the hand,” despite having the same shape as all other handles. This result can be explained by the fact that the resulting handle shape after it has been gripped may exhibit a different shape compared to the original due to the excessive deformation of the compliant interface material.

The 14% dense handle was rated the highest in terms of allowing for good task performance. A statistically significant difference was observed between the 14% and 6% dense handle and also the PLA stiff handle and the 6% dense handle. This suggests that medium- to high-density handles strike a balance between sufficient deformation for comfort and firmness for effective force transmission. The 6% handle’s lower rating suggests that it may hinder precise task execution due to excessive handle material deformation.

The comfort descriptor “Results in high peak contact pressures” revealed that the 6% dense handle resulted in the highest value; however, it was only statistically significant different from the 14% dense handle, which is in contradiction to the expected results. This can most likely be explained by the fact that due to the selected manual sawing task, the gripping forces were high. When observing the numerical results from contact pressure vs. bone vertical displacement and contact pressure vs. reaction force, the difference in contact pressure is evident, but not significant. Both values exceed the reported PDT and PPT values [17]. When observing the results from the anterior section, a larger area of the finger is in contact with the handle, hence triggering more touch receptors than with the stiffer 14% handle. Since both handles exceed the PPT and PDT values, the 6% dense handle most likely results in the feeling that it produces a higher peak contact pressure, despite resulting in lower values, as presented with the numerical results. Similar findings have also been reported before by Goonetilleke and Eng [42]. Therefore, these findings underscore the critical role of material stiffness in balancing tactile perception, comfort, and stability during gripping tasks.

The subjective ratings are consistent with our numerical findings, where medium-density handles (10% and 14%) demonstrated better performance in reducing contact pressure and maintaining stability. The numerical results confirmed that handles with 14% and 10% density showed a more uniform pressure distribution, reducing peak contact pressures and maintaining stability, which aligns with the subjective comfort ratings. Conversely, the 6% dense handle, while soft, deformed excessively under load, leading to instability and discomfort, as reflected in lower subjective ratings.

**Task dependency of preferred handle stiffness:** Unlike traditional stiff handle materials, which exhibit an exponential rise in contact pressure to increasing gripping forces, the cellular handle material demonstrates a distinct plateau phase, characterized by minimal changes in stiffness despite variations in applied force. This plateau-like behavior plays a crucial role in maintaining stability and minimizing excessive deformation under high loads, thus enhancing user comfort and tactile perception.

For instance, while soft deformable handles may offer lower contact pressures initially, their tendency to deform excessively under higher grasping forces can diminish stability and perceived comfort. This can be explained by the fact that users performing highly demanding tasks with soft deformable tool handles will grip the handle with higher force to compensate for the loss of stability, which has also been reported by Fellows and Freivalds [22]. The elastic region of the 6% dense handle is small, and hence the deformation plateau is reached already at low gripping forces. Therefore, almost all deformation of the handle during gripping can be attributed to the plateau region of the deformable handle material, which results in excessive deformation of the handle, ultimately lowering stability. Moreover, soft deformable handles with limited thickness can be deformed to the point that all cellular structures become deformed. Hence, the densification area of the cellular structure is already reached, which results again in a high rise in contact pressure on the hands.

In contrast, medium and hard deformable handles demonstrate more controlled deformation, with higher plateau levels, resulting in deformation of the handle material with higher grasping forces and hence improved stability and tactile perception. When observing the numerical results from contact pressure vs. displacement and reaction force at the finger reaction force of 35 N, it can be observed that with the 14% dense deformable handle, the deformation plateau is just reached, hence resulting in the lowest deformation of the handle and finger and handle vertical displacement. Interestingly, the results for the 10% dense handle show that the densification area has already been reached at the finger reaction force of 35 N, and the resulting peak contact pressure is almost the same as with the considerably stiffer 14% dense handle, but at higher deformation and displacement values.

Since no statistically significant differences have been found in any comfort descriptors and overall comfort between the 10% and 14% dense handles, it can be concluded that the gripping forces during the manual sawing task resulted in high values that are comparable to the higher end, like 35 N, as reported in the numerical results. The values of contact pressure at higher gripping forces are converging and change position after a 35 N reaction force.

Ultimately, the 14% dense handle was rated the best in overall comfort rating and was statistically significantly different from the PLA plastic handle and the 6% dense deformable handle, indicating that contact pressure distribution, as well as stability, has a significant influence on the perceived comfort rating. Previous research has demonstrated that variations in PDT and PPT values among different individuals can be attributed to the distinct geometries of their fingertip soft tissue and the underlying bone structures [15,17]. Individual differences in fingertip geometry, bone structure, and personal factors like past experience and tactile thresholds contribute to varied pressure responses and preferences. These variations likely explain the high standard deviation in comfort ratings and highlight the importance of involving the target demographic in refining bioinspired handle designs.

These findings align with biomimetic design principles, where adaptable stiffness and nonlinear deformation behavior are inspired by the mechanical properties of human soft tissue. Consistent with this approach, the present study confirms that tailored mechanical compliance of the tool handle, achieved through gyroid-based cellular metamaterial design, can offer substantial ergonomic benefits. Embedding such biomechanical intelligence into handheld tools represents a promising direction for improving haptic quality, reducing fatigue and mitigating discomfort in force-intensive manual tasks.

This study focused on a specific tool handle geometry and a single type of cellular metamaterial (gyroid structure), fabricated via FFF 3D printing. While our findings provide valuable insights into the ergonomic benefits of tunable stiffness, the generalizability of these results may be limited to similar applications. Future research could explore a wider range of tool geometries, alternative metamaterial architectures, and different manufacturing processes to further broaden the applicability of this bioinspired design approach. Additionally, other types of tools and products should be used in future studies to assess the broader impact of these ergonomic handle designs. Incorporating objective physiological measurements (e.g., muscle activity, localized tissue deformation) in future experiments could also provide a more comprehensive understanding of the hand–handle interaction.

## 5. Conclusions

This study demonstrated that integrating gyroid-based cellular metamaterials into handheld tool handles can significantly improve ergonomic and haptic performance. Handles with medium stiffness achieved the best balance between reducing peak contact pressure and maintaining grip stability, leading to the highest subjective comfort ratings across descriptors such as fit, ease of use, and overall stability. These findings support the application of biomimetic design principles, where controlled deformation and nonlinear stiffness emulate the mechanical behavior of human soft tissue. The plateau-like response of the cellular material under increasing load helps redistribute contact forces without compromising grip stability, thereby enhancing both comfort and tactile perception. Beyond improving comfort in force-intensive tasks, this approach has the potential to reduce fatigue and long-term strain injuries. Future work should explore other metamaterial architectures, conduct extended usability studies, and expand the concept to applications such as sports gear, assistive technologies, and robotic interfaces.

## Figures and Tables

**Table 1:** Material parameters used in numerical analysis, as derived from [23].

Component	Material Model	Parameters
Subcutaneous tissue	Ogden 3rd-Order	μ_1_ = −0.04895 MPa
		μ_2_ = 0.00989 MPa
		μ_3_ = 0.03964 MPa
		A_1_ = 5.511
		A_2_ = 6.571
		A_3_ = 5.262
		D_1_ = −4.2267 MPa^−1^
		D_2_ = 20.92 MPa^−1^
		D_3_ = 5.2194 MPa^−1^
Skin	Ogden 3rd-Order	μ_1_ = −0.07594 MPa
		μ_2_ = 0.01138 MPa
		μ_3_ = 0.06572 MPa
		A_1_ = 4.941
		A_2_ = 6.425
		A_3_ = 4.712
		D_1_ = −2.7245 MPa^−1^
		D_2_ = 18.181 MPa^−1^
		D_3_ = 3.1482 MPa^−1^
Distal phalanx	Rigid structure	(-)
Handle—PLA	Linear–elastic	E = 2952.8 MPa
		ν = 0.33
Handle—custom gyroid	Multilinear elastic	Defined by σ and ε points from experiment (Figure 1).

## Data Availability

Data available on request from the authors.
